# Urolithin-A Promotes CD8^+^ T Cell–mediated Cancer Immunosurveillance via FOXO1 Activation

**DOI:** 10.1158/2767-9764.CRC-24-0022

**Published:** 2024-05-03

**Authors:** Pierpaolo Ginefra, Helen Carrasco Hope, Yi-Hsuan Chiang, Sophie Nutten, Stephanie Blum, George Coukos, Nicola Vannini

**Affiliations:** 1 Department of Oncology, Ludwig Institute for Cancer Research Lausanne, University of Lausanne, Lausanne, Switzerland.; 2 Nestlé Health Science, Lausanne, Switzerland.

## Abstract

Naïve T cells are key players in cancer immunosurveillance, even though their function declines during tumor progression. Thus, interventions capable of sustaining the quality and function of naïve T cells are needed to improve cancer immunoprevention.

In this context, we studied the capacity of Urolithin-A (UroA), a potent mitophagy inducer, to enhance T cell–mediated cancer immunosurveillance.

We discovered that UroA improved the cancer immune response by activating the transcription factor FOXO1 in CD8^+^ T cell. Sustained FOXO1 activation promoted the expression of the adhesion molecule L-selectin (CD62L) resulting in the expansion of the naïve T cells population. We found that UroA reduces FOXO1 phosphorylation favoring its nuclear localization and transcriptional activity. Overall, our findings determine FOXO1 as a novel molecular target of UroA in CD8^+^ T cells and indicate UroA as promising immunomodulator to improve cancer immunosurveillance.

**Significance:**

Urolithin-A, a potent mitophagy inducer, emerges as a promising tool to enhance cancer immunosurveillance by activating the FOXO1 transcription factor in CD8^+^ T cells. This activation promotes the expansion of naïve T cells, offering a novel avenue for improving cancer immune response and highlighting UroA as a potential immunomodulator for bolstering our body's defenses against cancer.

## Introduction

Efficient cancer immune surveillance relies on the circulation of naïve CD8^+^ T cells between the blood and lymphatic vessels, and secondary lymphoid organs. Once primed by professional antigen-presenting cells in the lymph nodes, cancer-specific CD8^+^ T cells migrate into the tumor microenvironment (TME) to exert their cytolytic functions (1), where altered immunologic and metabolic cues interfere with CD8^+^ T-cell differentiation process and function (2, 3). Importantly, patients with cancer display both quantitative and qualitative defects in naïve T cells (4, 5). Therefore, the improvement of T-cell homeostasis is crucial for boosting cancer treatment and prevention. In this context, several studies have shown that naïve T cells homeostasis is controlled by cytokines such as IL7, IL15, and the interaction between the T-cell receptor (TCR) and the MHC (6). Among various transcription factors, the forkhead box O (FOXO) transcription family is fundamental in controlling the signaling for the maintenance of naïve T-cell homeostasis (7, 8). FOXO1 governs the expression of genes encoding homing molecules such as CD62 L and CCR7, and key regulators of survival pathways such as IL7 receptor (CD127; ref. 9). Interestingly, dysfunctional FOXO1 signaling has been associated with impaired homeostasis and proteostasis in T cells during aging (10).

Urolithin-A (UroA) is a first-class metabolite activator of mitophagy successfully tested to treat age-related disease in both preclinical (11) and clinical settings (12–15). UroA exhibits anti-inflammatory properties, and potent metabolic modulation properties, suggesting its application to attenuate or prevent the onset of several diseases, including Alzheimer's disease, diabetes, and nephrotoxicity (14). In addition, UroA acts on both innate and adaptive immune cells (14) by attenuating inflammatory state of macrophages or neutrophils (16, 17), inhibiting pathogenicity of Th17 cells (18) and boosting T cells anticancer activity in colon and pancreatic preclinical models (19, 20).

We have recently shown that UroA supplementation can reverse age-related dysfunction of immune cells by restoring mitochondrial fitness of hematopoietic stem cells (HSC) derived from old mice (21). In this study, we investigate the effect of UroA on T cells and its potential capacity to improve cancer immunosurveillance and demonstrate that UroA supplementation boosts cancer immunosurveillance by promoting FOXO1-transcriptional activity.

## Materials and Methods

### Mice and Cell Lines

C57BL/6J (RRID:IMSR_JAX:000664) females were purchased from Harlan Laboratories. OT1 TCR C57BL/6J (RRID:IMSR_JAX:000664), Mito-QC (MGI:5817670), and NOD scid gamma (NSG; RRID:IMSR_JAX:005557) mice were bred and maintained in-house. All animal experiments were performed in the animal facility in Epalinges at the University of Lausanne (UNIL) as approved by the veterinary authorities of the canton of Vaud, and performed in accordance with Swiss Federal Law (VD3572 and VD3684). B16-Ova (RRID:CVCL_WM78) melanoma cell line was obtained from Prof. George Coukos's Laboratory (UNIL) and were kept in culture DMEM (3133, Invitrogen) supplemented with 10% heat-inactivated FBS (GIBCO) and 1% penicillin/streptomycin (P/S), the YUMM 1.7 (RRID:CVCL_JK16) cell line was obtained from Ping-Chih Ho's laboratory, while the MC-38 (RRID:CVCL_B288) cell line was obtained from Prof. Pedro Romero's laboratory (UNIL) and kept in RPMI1640-Glutamax medium supplemented with 10% heat-inactivated FBS and 1% P/S. Cells were used with relatively low passage (<20) number and were not tested for *Mycoplasma*.

### Food Preparation for *In Vivo* Feeding

UroA diet was prepared by mixing custom synthesized UroA (Novalix) dissolved in DMSO with 2916 powder diet (Charles River) and air-dried into pellets in sterile conditions. The UroA dosage in the mix was calculated by considering the average mouse food intake per day (5 g food/mouse/day) for a calculated intake of 50 mg UroA/kg/day. Equivalent amount of DMSO was added in the control food. C57BL/6J (RRID:IMSR_JAX:000664), MitoQC, and NSG mice were fed with control or UroA diet for 4 weeks (or for the indicated time period if stated otherwise). In withdrawal experiments, mice were fed for 4 weeks with UroA-enriched or control food. Then, half of the mice were kept under the initial feeding condition, the other half received conventional food for 1 week before tumor engraftment. For FOXO inhibition experiments, mice were intraperitoneally injected with FOXO inhibitor AS1842856 (S8222, Selleckchem) at 10 mg/kg as previously described twice per week (22).

Weekly food consumption was monitored in all cages and no difference was found in the feeding behavior of mice upon UroA supplementation in the diet.

### Mitophagy Assessment

Mito-QC mice express a mCherry-GFP tandem protein targeted to the mitochondrial outer membrane. When mitochondria are degraded in the lysosomes, the acidic environment quenches the GFP but not the mCherry fluorescence and mitophagy can be assessed on the basis of unbiased mCherry/GFP ratio or by selected gating strategy (23, 24). When stated cells were treated with FOXO inhibitor AS1842856 (50 nmol/L; S8222, Selleckchem) or mitophagy inhibitor mDivi (10 µmol/L; HY-15886, MedChem Express).

### CD8^+^ T-cell Isolation and Activation and Culture Condition

Spleens were harvested and smashed through a 70-µm strainer. Upon red blood cell lysis CD8^+^ T cells were negatively selected using EasySep Mouse T cells Isolation Kit (19853 Stem Cell Technologies) according to the manufacturer's protocol. Isolated T cells were then resuspended in RPMI Glutamax supplemented with 10% heat-inactivated FBS 1% P/S, 1% sodium pyruvate, 0.1% 2-mercaptoethanol and stimulated with anti-CD3/CD28 dynabeads (2:1 = beads: T-cell ratio; 11453D, Thermo Fisher Scientific), 50 IU/mL of murine IL2 (212-12, PeproTech). Starting from the third day of culture, beads were removed and the cells were maintained at a concentration of 5 × 10^5^/mL in presence of human IL15 (Miltenyi Biotec, 130-095-765) and human IL7 (Miltenyi Biotec, 130-095-362) at 10 ng/mL or in presence of 50 IU/mL of murine IL2. UroA (SML1791, Sigma-Aldrich) was supplemented at 5 µmol/L (or at different concentration if stated otherwise) from day 3 and refreshed with cytokines at days 5, 7, and 9. For chronic stimulation experiments, control and UroA-treated cells were as described previously (25). Briefly, CD8^+^ T cells were simulated with 10 ng/mL Ova peptide for 5 days before further analysis. In FOXO1 inhibition experiment, CD8^+^ T cells were treated for 48 hours with FOXO1 inhibitor AS1842856 (50 nmol/L; S8222, Selleckchem). To assess FOXO1 phosphorylation, CD8^+^ T cells were treated for 4 hours with UroA (5 µmol/L). For MitoQC T-cell culture, splenocytes from spleen of MitoQC mice were stimulated for 3 days in presence of CD3 (3 µg/mL) and CD28 (2 µg/mL) antibodies. Starting from the third day of culture, the cells were washed and maintained at a concentration of 5 × 10^5^/mL with human IL15 (Miltenyi Biotec, 130-095-765) and human IL7 (Miltenyi Biotec, 130-095-362) at 10 ng/mL or in presence of 50 IU/mL of murine IL2 for the indicated time. In mitophagy inhibition experiments, CD8^+^ T cells were treated for 48 hours with mDIVI (10 µmol/L; HY-15886, MedChem Express). For phospho-Akt experiments, CD8^+^ T cells were treated with UroA (5 µmol/L) before being stimulated for 30 minutes with CD3 (3 µg/mL) and CD28 (2 µg/mL) antibodies.

### Adoptive T-cell Transfer Experiment

Briefly, 1 × 10^5^ B16-Ova cells were subcutaneously injected in the right flank of 7–9 weeks old mice. After 10 days, mice were randomized to have comparative tumor volumes and received 5 Gy irradiation followed by two rounds of intravenous injection of 1 × 10^6^ T cells at days 11 and 14 after tumor engraftment. Mice were monitored three times a week, and tumor length (*L*; greatest longitudinal measurement) and width (*W*; greatest transverse measurement) were measured with a caliper *r* in a blinded manner. Tumor volumes (*V*) were calculated using the formula: *V*  =  (*L*  ×  *W*^2^)/2. Mice were euthanized at endpoint by CO_2_ and, where indicated, tumors, spleens, and lymph nodes were collected. For *ex vivo* analysis, at terminal point solid tumor mass was excised from the mice, weighed, cut into small pieces with a scalpel, passed through 70 µm pore cell strainers and centrifuged for 5 minutes at 1,500 rpm to pellet the cells. The cells were then resuspended in PBS and a Ficoll gradient was performed to eliminate dead cells and tumoral debris by centrifugation at 2,000 rpm for 20 minutes with acceleration 2 and brake 7. Upon extensive washes, the cells were used for flow cytometric analysis.

### Feeding Experiments and Tumor Challenge

Briefly, 1 × 10^5^ B16-Ova cells or 1 × 10^6^ MC-38 cells or 10 × 10^5^ YUMM 1.7 cells were subcutaneously injected in the right flank of 7–9 weeks old C57BL/6J or NSG mice previously feed with control or UroA (for 4 weeks, unless differently stated). For rechallenge experiments, 1 × 10^6^ MC-38 cells were subcutaneously injected in the left flank. For immune depletion experiment, 1 × 10^5^ B16-Ova cells were subcutaneously injected in the right flank of 7–9 weeks old C57BL/6J mice previously feed with control or UroA. CD8^+^ T cells were depleted by administering five times 300 µg of depleting antibody (clone 2.43 Bio X Cell) every 2 days after tumor engraftment. For checkpoint blockade experiment, 1 × 10^5^ B16-Ova cells were subcutaneously injected in the right flank of 7–9 weeks old C57BL/6J mice previously feed with control or UroA followed by four rounds of 100 µg anti-PD1 (clone BE0146 Bio X Cell).

### Flow Cytometry

For analysis of surface markers, T cells were stained in FACS buffer (PBS containing 2% FBS and 2 mmol/L Ethylenediaminetetraacetic acid) with antibody mixture on ice at 4 degrees for 30 minutes. For intracellular staining, T cells were fixed and permeabilized with Fix/Perm buffer according to manufacture protocol. The antibodies used were: CD3 (145-2C11), CD8 (53.6.7), CD44 (IM.781), CD62 L (Ma-ƒMel-14), TCF-1 (C63D9), PD-1 (29F.1A12), TIM3 (RMT3-23), PGC1-α (D-5), CD45.2 (104), CD4 (RM4-4), FOXp3 (FJK-16S),NKp46 biotin (polyGaM), CD19 (ID3), CD11b (M1/70), CD11c (N418), Ly6C (HK1.4), Ly6G (1A8-Ly6g), F4/80 (BM8), FOXO1 (C29H4, Cell Signaling Technology), pFOXO1 (9461, Cell Signaling Technology), pAKT (4060, Cell Signaling Technology), and secondary anti rabbit 488 (4412S, Cell Signaling Technology), and streptavidin PeCy7.

For distinguishing live versus dead cells, the Live/Dead Fixable near red kit was used. Counting beads were used to assess cell number. For phospho-staining, after cell surface staining the cell were fixed in paraformaldehyde 4% for 10 minutes and then permeabilized in cold methanol for 15 minutes at 4 degrees before incubation with primary antibody.

In particular, for TME analysis we adopted a previously described protocol and gating strategy (26). Briefly, tumor was excised from the mice, weighed, cut into small pieces with a scalpel and digested in RPMI with 2% FBS, DNAseI (1 µg/mL), and collagenase (0.5 mg/mL) for 45 minutes at 37 degrees. The digested samples were passed through 70 µm pore cell strainers. Single-cell suspension was incubated with Live Dead Dye followed by FC blocking step performed with FC blocking solution prepared by the flow cytometry facility. Then cells were stained as previously described with desired antibody mix.

For mitochondrial analysis, T cells were stained with 20 nmol/L Tetramethylrhodamine-Methyl Ester-Perchlorate (T668, Thermo Fisher Scientific) and 100 nmol/L Mitotracker Green FM (M7514, Invitrogen) for 30 minutes at 37°C for mitochondrial membrane potential and mass, respectively.

The samples were acquired with the LSRII or Cytoflex machines at the UNIL Flow Cytometry Facility.

### Real-time Quantitative PCR

Total RNA was extracted from UroA-treated T cells using RNeast Mini Kit (74104, Qiagen), following manufacturer's instructions. RNA was retrotranscribed to cDNA with PrimeScript cDNA Synthesis Kit (6110A, Takara). qRT-PCR reactions were performed using 1.5 µL of cDNA, 5 µL of Power Syber Green mastermix (Applied Biosystems) and 200 nmol/L of primers and analyzed on the 7900HT system (Applied Biosystems). The relative expression of *Actb* was used to normalize gene expression across samples. The following primers were used:

*CCR7*:

FW: CAGGCTTCCTGTGTGATTTCTACA
RV: ACCACCAGCACGTTTTTCCT

*CD62L*:

FW:ACGGGCCCCAGTGTCAGTATGTG
RV:TGAGAAATGCCAGCCCCGAGAA

*KLF2*:

FW: GCGTACACACACAGGTGAGA
RV: GCACAAGTGGCACTGAAAGG

*S1P1*:

FW: GTG TAG ACC CAG AGT CCT GCG
RV: AGC TTT TCC TTG GCT GGA GAG

*Actb*:

FW: GAGACCTTCAACACCCC
RV: GTGGTGGTGAAGCTGTAGCC

### Microscopy

FOXO1 localization was analyzed via confocal microscopy. UroA-treated T cells were stained as previously described for intracellular staining with FOXO 1 antibody (C29H4, Cell Signaling Technology) followed by staining with secondary antibody anti-rabbit 488(4412S, Cell Signaling Technology). Then the cells were adhered to glass slides via Cell-TAK (354241, Corning) coating and stained with DAPI before mounted with DABCO mounting medium. Cells were imaged with LSM800. Confocal images were analyzed using ImageJ (RRID:SCR_003070) software. DAPI staining was used to generate a mask or region of interest and FOXO1 signal was quantified in the DAPI region.

### Quantification and Statistical Analysis

All statistical analyses were performed using GraphPad Prism. Numbers of samples and experiments are stated in each figure together with the statistical test adopted. Data are shown as mean ± SEM. Only significant comparisons are described, values of *P* < 0.05 were considered significant. All the experiments have been replicated at least once.

### Data and Materials Availability

All data and materials are available to any researcher for purposes of reproducing or extending the analyses.

## Results

### Preexposure to UroA-enriched Diet is Sufficient to Delay Tumor Growth

**FIGURE 1:**
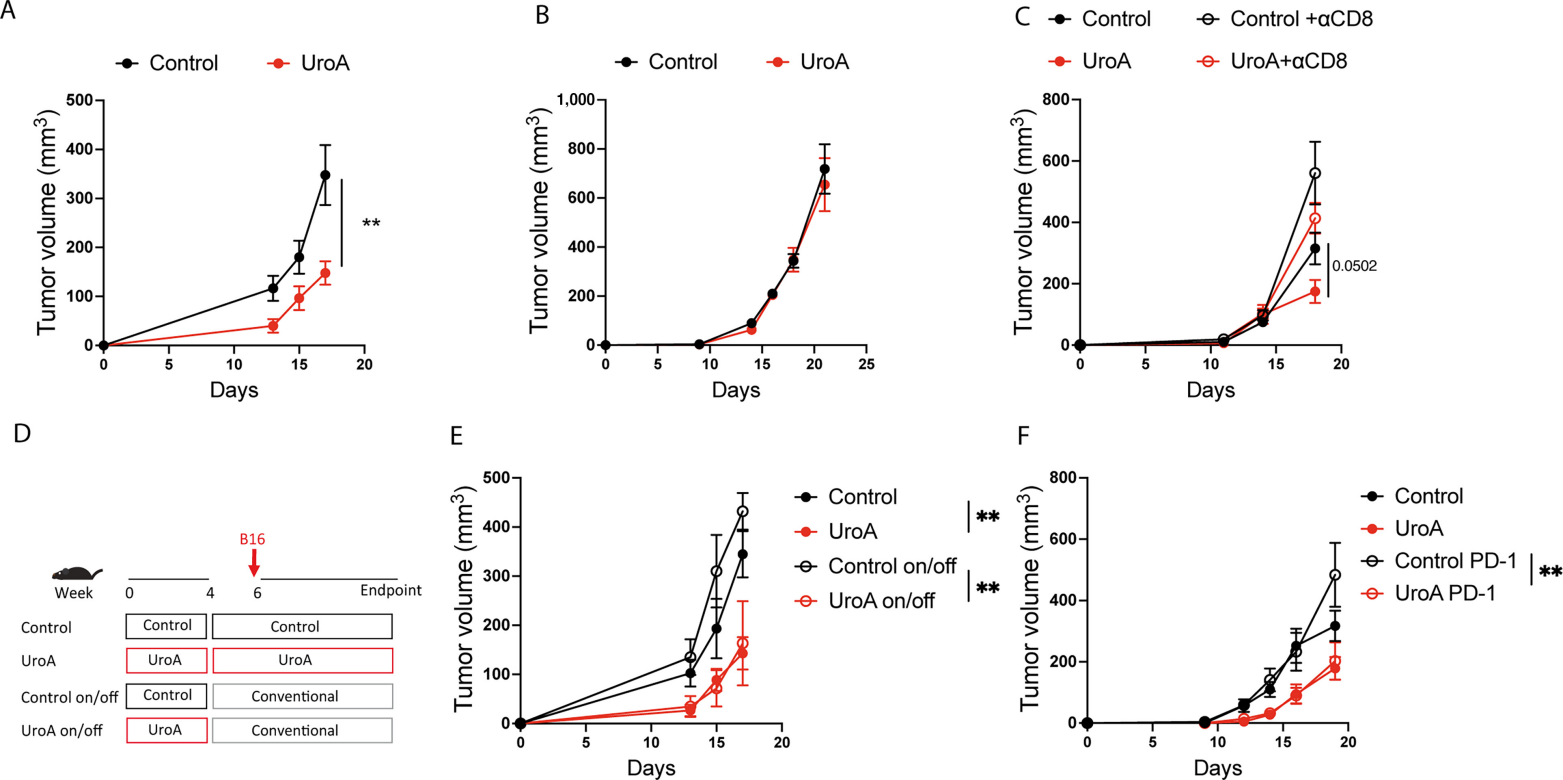
UroA supplementation slows tumor progression in immunocompetent mice. **A,** Analysis of tumor growth rate of B16 tumors cells injected in immunocompetent C57/Bl6 mice fed for 4 weeks with UroA-enriched food before tumor cells injection. **B,** Analysis of tumor growth rate of B16 tumors cells injected in immunodeficient NSG mice fed with control or UroA-enriched diet for 4 weeks before tumor cells injection. **C,** Analysis of tumor growth rate of B16 tumors implanted in mice as in A and treated with or without anti-CD8 antibody. **D,** Schematic representation of *in vivo* feeding experiment followed by withdrawal condition. Mice were fed for 4 weeks with UroA-enriched or control food. Half of the mice were kept under the initial feeding condition, the other half received conventional food. One week after, B16 cells were subcutaneously injected. **E,** Analysis of tumor growth rate in H. **F,** Tumor growth rate of B16 tumors in mice as in A and treated with anti PD-1 therapy. Data are mean ± SEM. Each dot represents a biological replicate. In **A,** control = 6 mice, UroA = 7 mice. In **B,** control = 5 mice, UroA = 5 mice. In **C,** control = 6 mice, UroA = 7mice, control+CD8 = 6 mice, UroA+CD8 = 6 mice. In **E,** control and UroA = 7 mice, on/off condition = 5 mice. In **F,** each group has 6 mice. Data were analyzed by two-sided Student *t* test or by ANOVA test followed by multiple comparison test (**, *P* < 0.01). Representative results of two independent experiments.

To assess UroA impact on immune function and cancer response, mice were fed with UroA diet for 4 weeks and then subcutaneously injected with B16 melanoma cell line (Supplementary Fig. S1A). Notably, immunocompetent C57BL6 mice supplemented with UroA exhibited reduced tumor growth (Fig. 1A), while this effect was absent when B16 melanoma cells were engrafted in immunodeficient NSG mice (Fig. 1B). Similarly, the depletion of CD8^+^ T cells in immunocompetent mice abolished the UroA-mediated tumor control (Fig. 1C). To demonstrate that the observed effect of UroA was not specific to a particular cancer type, we monitored the tumor growth in mice engrafted with a different tumor cell line, MC38 colon carcinoma cells, and fed with UroA-enriched diet. Similarly, to what observed in the B16 model, UroA fed mice displayed reduced tumor growth (Supplementary Fig. S1B), a phenomenon not observed when MC38 cells were engrafted in immune deficient NSG mice (Supplementary Fig. S1C). Importantly, tumor-free survivor mice maintained tumor control capacity upon rechallenged with new MC38 engraftment (Supplementary Fig. S1D), indicating the instauration of immunologic antitumor memory. Similarly, UroA reduced the growth of subcutaneously engrafted YUMM 1.7 melanoma cells (Supplementary Fig. S1E). Overall, these data reveal the UroA capacity to control tumor growth is mediated by immune system, particularly by CD8^+^ T cells.

However, when we analyzed the tumor immune infiltrate and CD8^+^ T-cell state in the TME of non-immunogenic B16 melanoma tumor, we did not observe changes in the percentage of both lymphoid/myeloid immune infiltrate or the exhaustion state and number of CD8^+^ T cells in mice fed with UroA (Supplementary Fig. S1F–S1I). Recent studies have indicated that the antitumor effect of UroA is mediated via the expansion of T stem cell memory and naïve T-cell compartment within the TME of colon (19) and pancreatic (20) tumors. Therefore, we analyzed the phenotype of CD8^+^ T cell in the TME of B16 and YUMM 1.7 tumors. In the TME of the two melanoma models, we observed similar frequency of central memory and effector memory CD8^+^ T cells (Supplementary Fig. S1J). UroA supplementation did not expand naïve T cells, which comprises the T stem cell memory compartment, in the TME of our melanoma models (Supplementary Fig. S1J).

Interestingly, mice fed with UroA-enriched diet and engrafted with B16 melanoma cells and treated with anti-PD-1 immunotherapy did not display better tumor control (Fig. 1F), confirming that UroA supplementation did not modulate CD8^+^ T-cell state within the TME in the B16 melanoma. This observation suggests that CD8-dependent tumor control driven by UroA supplementation derives from a preacquired host immune condition more refractory to tumor development. To test this hypothesis, we compared tumor growth of mice fed for 4 weeks with UroA-enriched diet, followed by withdrawal 1 week before tumor engraftment (on/off condition), with that of mice receiving UroA-enriched diet for all the duration of the experiment (Fig. 1D). Strikingly, on/off condition mimicked the tumor control observed in mice continuously fed with UroA-enriched diet (Fig. 1E), suggesting that UroA supplementation during tumor progression was dispensable. In fact, when we monitored tumor growth in mice that started UroA supplementation at the day of tumor engraftment, we did not observe better tumor control (Supplementary Fig. S1K), indicating that the UroA-mediated tumor control is exerted through the establishment of an immune barrier hindering cancer development and not by directly interfering with the immune infiltrate of the TME. Altogether, these data suggest that long-term UroA supplementation is necessary to predispose an immune environment in the host refractory to cancer development without directly affecting the immune infiltrate within the TME.

### UroA Promotes Naïve T-cell Formation *In Vivo* and Central Memory Formation *In Vitro*

**FIGURE 2:**
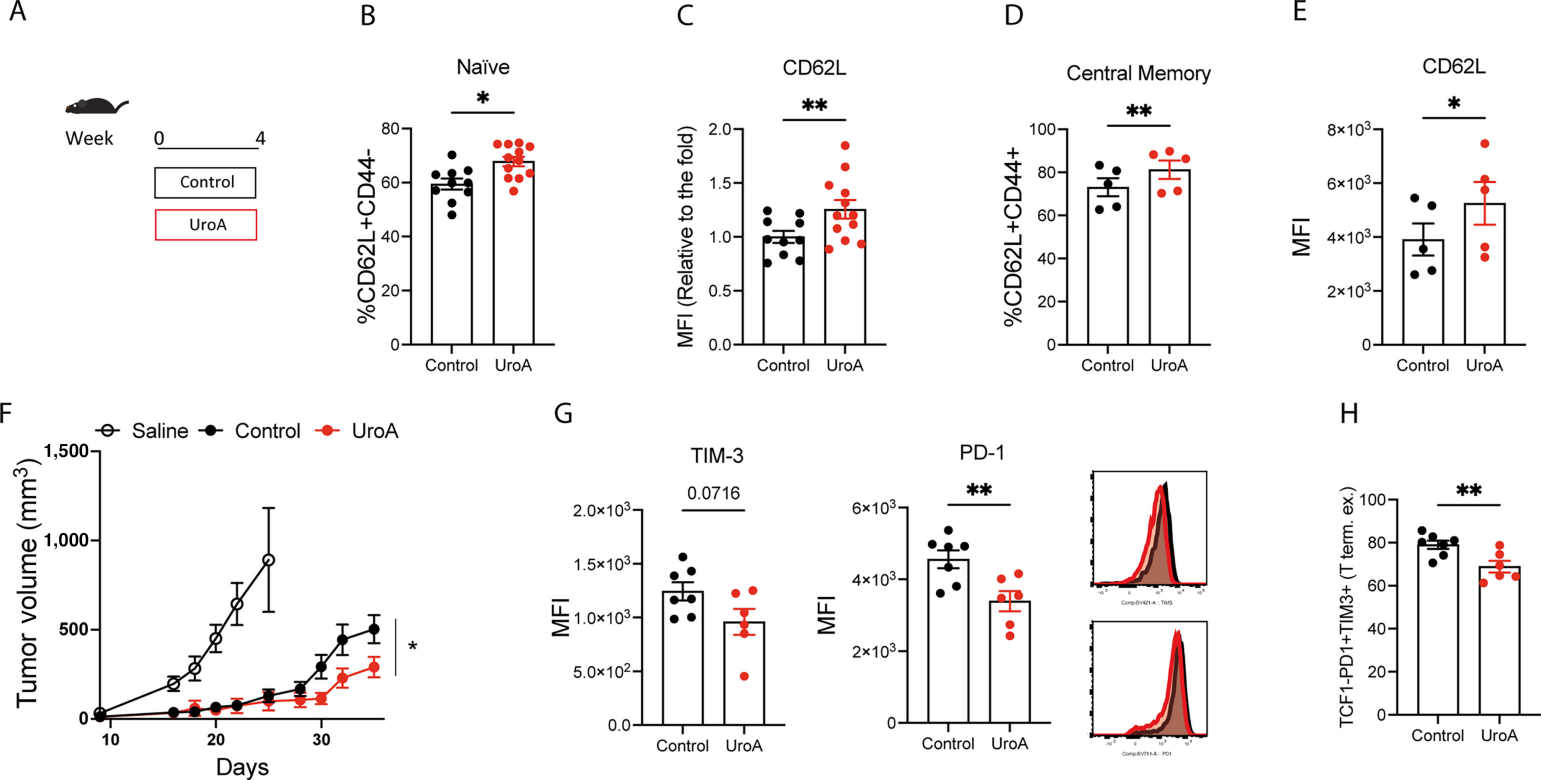
UroA promotes naïve T-cell expansion *in vivo* and memory formation *in vitro.***A,** Schematic representation of *in vivo* feeding experiment. **B,** Frequency of naïve CD8^+^ T cells (CD62L^+^CD44^−^) isolated from the spleen of mice fed with control or UroA-enriched diet. **C,** Expression level of CD62 L in CD8^+^ T cells from B, values are normalized to the control. **D,** Frequency of central memory (CD62L^+^CD44^+^) in CD8^+^ T cells treated for 4 days with 5 µmol/L UroA in IL15/7 condition *in vitro*. **E,** Expression level of central memory marker CD62 L in C. **F,** Analysis of tumor growth rate of B16-Ova tumor-bearing mice adoptively transferred with no cells (saline = 13 mice), control OT-1 CD8^+^ T cells (control = 10 mice) and UroA-treated OT-1 CD8^+^ T cells (UroA = 13 mice). **G,** Quantification of expression level of TIM-3 and PD-1 in TILs from F measured (control = 7, UroA = 6). **H,** Frequency of terminally exhausted T cells (TCF1^−^PD1^+^TIM3^+^) from G. Data are mean ± SEM. Each dot represents a biological replicate. In **B** and **C,** control *n* = 10 mice, UroA = 12 mice. In **D** and **E,** sample size *n* = 5. Data were analyzed by two-sided Student *t* test (*, *P* < 0.05; **, *P* < 0.01). Representative results of two independent experiments or two pooled experiments.

The lack of a direct effect of UroA supplementation on the immune infiltrate of the TME prompted us to analyze T-cell profiles in lymphoid organs of mice supplemented with UroA to better understand its mechanism of action (Fig. 2A). UroA supplementation increased the percentage of naïve CD8^+^ T cell in both spleens and lymph nodes (Fig. 2B; Supplementary Fig. S2A), an effect that was also observed in the CD4^+^ population (Supplementary Fig. S2B). Importantly, naïve CD8^+^ T cells displayed increased L-selectin (CD62L) expression (Fig. 2C; Supplementary Fig. S2A), an adhesion molecule playing fundamental role in T-cell homing in lymphoid organs. Subsequently, we tested the direct effect of UroA on T cells by exposing OT-1 CD8^+^ T to UroA *in vitro.* Activated T cells were treated for 7 days with 5 µmol/L UroA to match the current expansion protocol for adoptive cell therapy (ACT) products (27, 28) and avoid UroA toxicity (19) when supplemented at high concentration (>10 µmol/L) or during T-cell activation step (e.g., CD3/CD28 activation beads; Supplementary Fig. S2C and S2D). Similarly, to what observed in the *in vivo* conditions, OT-1 CD8^+^ T cells expanded in memory (IL15/7) polarizing conditions increased CD62 L expression and promoted memory-like T-cell formation when cultured in the presence of UroA (Fig. 2D and E; Supplementary Fig. S2E). Interestingly, increased CD62 L expression was also observed when OT-1 CD8^+^ T cells were expanded in effector polarizing condition (IL2; Supplementary Fig. S2E–S2G). The acquisition of memory-stem like phenotype is crucial but not sufficient to predict T-cell antitumor capacity. The TME imposes important metabolic and functional changes to T cells, which hamper their antitumor capacity and leads to the acquisition of exhausted state and consequent expression of specific markers such as PD-1, TIM-3 (2). Indeed, chronically stimulated T cells upregulate the expression of exhaustion markers (29); however, preexposure to UroA significantly reduced the expression levels of the exhausted markers (Supplementary Fig. S2H and S2I). To finally prove that UroA exposure was sufficient to boost CD8^+^ T-cell antitumor activity, OT-1 T cells treated with UroA were transferred into B16-Ova tumor-bearing mice. Importantly, UroA-treated OT-1 T cells had better tumor control capacity (Fig. 2F). Analysis of immune infiltrate revealed that tumor-infiltrating lymphocytes (TIL) derived from UroA-treated T cells expressed lower level of PD-1 and TIM3 (Fig. 2G). Consequently, the proportion of terminally exhausted TILs was reduced (Fig. 2H; refs. 2, 30). The higher level of CD62 L expression on target-specific CD8^+^ T cell favors their migration in the draining lymph nodes (31, 32) and confers protective immunity against infection and cancer (33, 34). Interestingly, our analyses revealed that UroA treatment expands T cells retaining central memory phenotype in the draining lymph nodes (Supplementary Fig. S2J). Our data suggest that UroA improves CD8^+^ T-cell function by sustaining CD62 L expression and maintaining central memory phenotype.

### UroA Induces the Expression of FOXO1 Target Genes in CD8^+^T Both *In Vitro* and In *Vivo*

**FIGURE 3:**
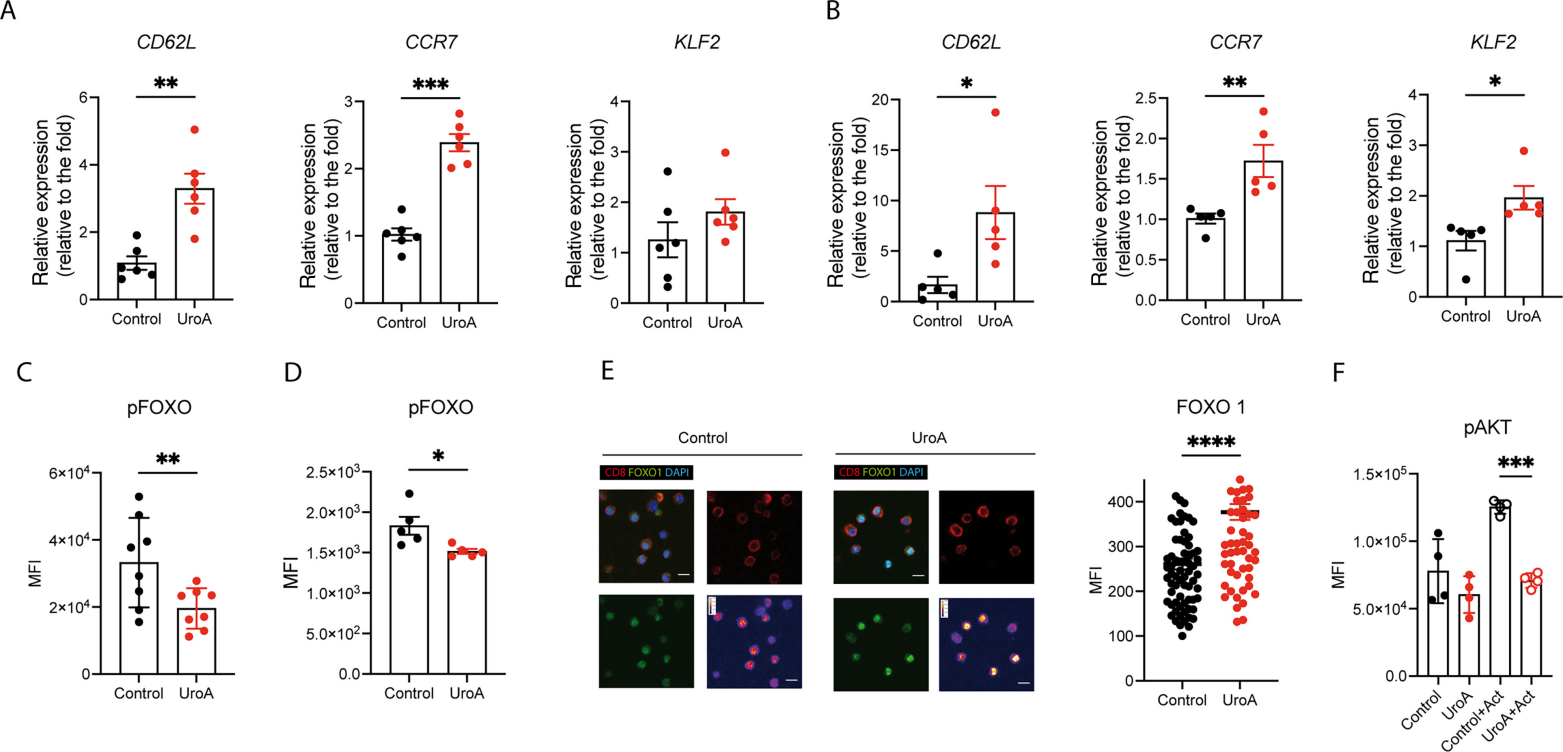
UroA induces FOXO1 transcriptional activity in CD8^+^ T cell. **A,** Quantification of FOXO1 target genes in CD8^+^ T cells culture with 5 µmol/L UroA in IL15/7 condition *in vitro*. Values are expressed as fold change compare with control condition. **B,** Analysis of FOXO1 target genes expression levels in CD8^+^ T cells isolated from mice fed with control or UroA-enriched food for 4 weeks. Values are expressed as fold change compare with control condition. **C,** FOXO1 phosphorylation in CD8^+^ T cells treated for 24 hours with 5 µmol/L UroA *in vitro*. **D**, FOXO1 phosphorylation in CD8^+^ T isolated from mice fed with control or UroA diet. **E**, Representative confocal images of CD8^+^ T cells stained with FOXO1 (green) CD8 (red), and DAPI (blue) under the indicated culture condition (scale bar, 5 µm) *in vitro* (UroA = 5 µmol/L). The LUT images represent FOXO1 intensity staining (left). Quantification of nuclear FOXO1 in control and UroA (5 µmol/L) treated CD8^+^ T cells) *in vitro* (right). **F,** Akt S473 phosphorylation in CD8^+^ T cells treated for 24 hours with 5 µmol/L UroA *in vitro* and then stimulated with CD3/CD28 antibodies for 30 minutes (Act condition). Data are mean ± SEM. Each dot represents a biological replicate. In **A,** sample size *n* = 6. In **B,** sample size *n* = 5. In **C,** sample size *n* = 8. In **D,** sample size *n* = 5. In **E,** control = 72, UroA = 68. In F, sample size *n* = 4. Data were analyzed by two-sided Student *t* test (*, *P* < 0.05; **, *P* < 0.01; ***, *P* < 0.001; ****, *P* < 0.0001). Representative results of at least two independent experiments or two pooled experiments.

The FOXO family proteins regulate several aspects of T-cell biology, including trafficking, naïve homeostasis, differentiation, and effector and memory response (7, 8). Intriguingly, the UroA-driven improvement of CD8^+^ T-cell function is associated with a higher expression of the memory marker CD62 L (Fig. 2C and E), a known FOXO1 target gene. Moreover, both *in vitro* and *in vivo* supplementation of UroA increased the expression levels of FOXO1 target genes *CD62L*, *CCR7,* and *KLF2* in CD8^+^ T cells (Fig. 3A, and B; refs. 9, 35, 36). Importantly, UroA effect on FOXO1 target genes was observed also in the effector polarizing condition (IL2), where UroA increased the expression of FOXO1 downstream genes *CCR7* and *S1P1* (Supplementary Figs. S2G and S3A; ref. 37). The UroA-mediated effect on FOXO1 target genes was not a consequence of changes in FOXO1 expression (Supplementary Fig. S3B) but rather resulted from reduced FOXO1 phosphorylation (Fig. 3C and D), which prevents its nuclear localization and DNA binding (7). Indeed, confocal imaging analysis showed higher localization of FOXO1 in the nucleus of UroA-treated cells (Fig. 3E; Supplementary Fig. S3C). One of the major mediators of FOXO1phosphorylation is Akt, which functions as damper of FOXO1 transcriptional activity (38). Interestingly, UroA has been shown to target and to block PI3K/Akt pathway in pancreatic cancer cell line *in vitro* (39) and to reduce the level of phosphorylated AKT in pancreatic tumors *in vivo* (20). We thus treated T cells with UroA and tested its capacity to reduce Akt phosphorylation. In line with previous observations, UroA treatment reduced the levels of phosphorylated Akt in T cells (Fig. 3F), indicating Akt as potential mediator of UroA effect on FOXO1 in T cells.

Altogether, these data suggested that UroA effect on CD8^+^ T cells is in part mediated by FOXO1 transcriptional activity.

### UroA-induced Upregulation of FOXO1 Transcriptional Activity is Mitophagy Independent

**FIGURE 4:**
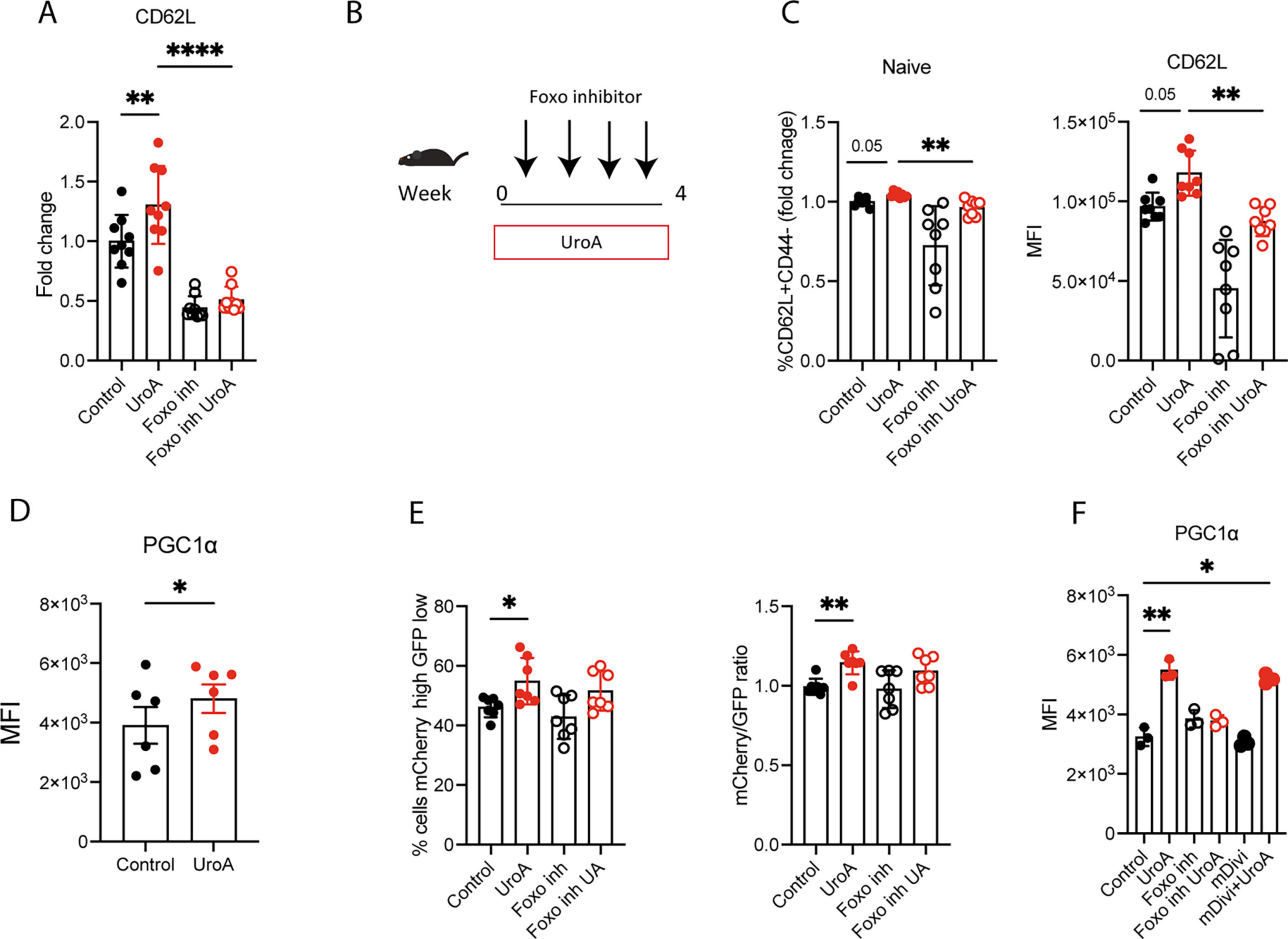
UroA-induced FOXO1 transcriptional activity is mitophagy independent. **A,** Quantification of CD62 L expression level in CD8^+^ T treated with UroA (5 µmol/L) and in presence of FOXO1 inhibitor (Foxo inh 50 nmol/L) for 48 hours *in vitro*. Values are expressed as fold change compare with control condition. **B,** Schematic representation of FOXO1 inhibition *in vivo* analyzed in C. **C,** Quantification of naïve CD8^+^ T population isolated from mice fed with UroA diet and treated with Foxo inh. Values are expressed as fold change compare with control condition (left). Quantification of CD62 L expression level in CD8^+^ T isolated from mice fed with UroA diet and treated with Foxo inh (right) for 48 hours *in vitro*. **D,** Expression level of PGC1α in CD8^+^ T treated with UroA (5 µmol/L) for 48 hours *in vitro*. **E,** Quantification of Mito-QC CD8^+^ T cells engaging mitophagy under indicated condition *in vitro* for 24 hours (UroA 5 µmol/L, Foxo inh 50 nmol/L). Quantification of mCherry/GFP ratio from E (right). **F,** Expression level of PGC1α in CD8^+^ T cells cultured in presence of Foxo inh (50 nmol/L) or mitophagy blocker (mDivi 10 µmol/L) for 48 hours *in vitro*. Data are mean ± SEM. Each dot represents a biological replicate. In **A,** sample size *n* = 9. In **C,** sample size *n* = 8. In **D,** sample size *n* = 6. In **E,** sample size *n* = 7. In **F,** sample size *n* = 3. Data were analyzed by two-sided Student *t* or ANOVA test followed by multiple comparison test (*, *P* < 0.05; **, *P* < 0.01; ****, *P* < 0.0001). Representative results of at least two independent experiments or two pooled experiments.

To confirm that UroA-induced upregulation of CD62 L was dependent on FOXO1 transcriptional activity, we used the FOXO1 inhibitor AS1842856 (hereafter named Foxo inh; ref. 40). FOXO1 inhibition reversed the UroA-induced overexpression of CD62 L in CD8^+^ T cell *in vitro* (Fig. 4A; ref. 41). Similarly, FOXO inhibition hindered UroA-dependent naïve T-cell expansion and CD62 L upregulation *in vivo* (Fig. 4B and C).

UroA has been shown to reduce mitochondrial potential and induce mitophagy in CD8^+^ T cells, potentially overcoming the dysfunctional exhausted state induced by damaged mitochondria (19). To explore whether the increased FOXO1 transcriptional activity was dependent on UroA mitophagy activity, we first tested the capacity of UroA to induce mitophagy in CD8^+^ T cells adopting the mitophagy reporter MitoQC mice, which have been previously employed to investigate UroA-induced mitophagy (21, 42). Our findings revealed evidence of mitophagy induction and mitochondrial depolarization in UroA-treated CD8^+^ T cells when cultured in memory polarizing condition (Supplementary Fig. S4A–S4C). On the contrary, we did not observe mitophagy induction when UroA was supplemented in effector polarizing condition (Supplementary Fig. S4D).

Moreover, UroA-treated T cells expressed higher level of PGC1α (Fig. 4D) and displayed higher mitochondrial mass (Supplementary Fig. S4E) in presence of IL15/7. Because PGC1α is a master regulator of mitochondrial biogenesis and a known target of FOXO1 (43, 36), we tested whether UroA-mediated regulation of mitochondrial dynamics was FOXO1 dependent. Interestingly, FOXO1 inhibition did not revert mitophagy processes in UroA-treated CD8^+^ T cells as shown by both gating strategy and ratiometric analysis (Fig. 4E). On the contrary, treatment with the mitophagy inhibitor mDIVI hindered UroA-induced mitophagy (Supplementary Fig. S4F; ref. 44), but did not affect the expression of FOXO1 targets like PGC1 and *CCR7*, which were instead sensitive to FOXO inhibition (Fig. 4F; Supplementary Fig. S4G). Overall, our results demonstrate that UroA effect on CD8^+^ T cells is mediated by FOXO1 transcriptional activity independently from mitophagy induction and reveals FOXO1 as novel non-mitochondrial UroA target.

## Discussion

The immune system is the main barrier protecting the organism against cancer insurgence and progression. In this context, T cells represent the most efficient and exploited immune cell type in cancer immunotherapy and serve as guardian in cancer immunosurveillance. Naïve T cells are crucial for preventing the onset of cancer, with their function tightly linked to the cellular metabolic state. Our previous work demonstrated that 3 months exposure to UroA improves CD8^+^ T cells response against viral infection in old mice (21), where, part of the effect, derived from an enhanced mitochondrial fitness in the HSC compartment, contributing directly to the generation of a better T-cell pool. However, our work did not exclude the possible direct effect of UroA on the T-cell compartment.

UroA has been described to possess immunomodulatory properties (14), which has been mostly investigated in the context of cancer immune response (19, 20). In the current work, we investigate the effect of UroA supplementation in CD8^+^ T cells unrevealing its capacity to promote CD8^+^ T cell–dependent cancer immunosurveillance. Our study demonstrates that UroA supplementation leads to the expansion of naïve CD8^+^ T-cell pool in the spleen and in the lymph nodes *in vivo* (45, 46), and delays cancer onset. In accordance with previous observations (4, 5), our results suggest that UroA supplementation enhance immunosurveillance and tumor control through the expansion of naïve T-cell pool.

Although UroA supplementation has been reported to improve immune checkpoint blockade (ICB) therapy in colorectal and pancreatic cancer models by boosting TIL function (19, 20), in our experimental condition we did not observe significantly changes neither in response to ICB nor TILs exhaustion state. This discrepancy might be attributed to the different characteristics of the B16 model, known to be refractory to ICB and poorly immunogenic. However, when we tested a more immunogenic tumor model, the YUMM 1.7 melanoma model, despite observing slower tumor progression, we did not reveal expansion of naïve T cells in the TME as reported previously. Work from Mehra and colleagues has shown that UroA-mediated tumor control is achieved via inhibition of intratumorally fibrosis, reduction of tumor-associated macrophages (M2-like) and decrease immunosuppressive cytokines in the TME of pancreatic ductal adenocarcinoma (20). Mehra and colleagues have reported that UroA administration supports the expansion of naïve T cells (both CD8^+^ and CD4^+^) in the TME (20). Therefore, the UroA immunomodulation might vary depending on the tumor type. Even though we did not observe naïve T cells expansion in the TME of B16 and YUMM 1.7 tumors upon UroA supplementation, we detected the expansion of the naïve T-cell pool in the spleen and in the lymph nodes, an important immunologic aspect that has not been reported previously. However, we cannot completely exclude the involvement of other immune populations within the TME in the UroA-mediated cancer immune response.

The same metabolic modulator can shape differently the immune landscape of TME depending on the dosage adopted among the studies. For example, the natural polyamine spermidine has been shown to promote immunosuppression through the expansion of regulatory T cells and M2 macrophages (47, 48). On the opposite, another study has reported that spermidine supplementation increased inflammation and improved antitumor activity in mice, without affecting regulatory T cells and M2 macrophages population (48). Importantly, in this study, the spermidine dose was 15- to 25-fold lower compared with the other ones (48).

In our work, the timing and the dosage of UroA supplementation differed from previous reports. We fed the mice for at least 4 weeks before tumor engraftment, while Denk and colleagues for example, provided UroA upon tumor formation (19), while Mehra and colleagues opted for a gavage administration after tumor formation (20). The chosen timeframe of 1 month supplementation aligns with previous studies and excludes a possible contribution deriving from HSCs (15, 49). In our *in vivo* experiments, the UroA amount is 9-fold lower that the one adopted by Denk and colleagues corresponding to the common dose of 50 mg/kg/day which is similar to the one chosen by Mehra and colleagues (20 mg/kg/day) and adopted in clinical trials (11, 15, 19, 20). This indicates that optimal dosage and timing of UroA supplementation should be optimized in a case-specific manner. However, despite the differences in shaping the TME, our studies along with the work of Denk and Mehra indicate a potent immune-mediated tumor control upon UroA supplementation.

In fact, short *in vitro* exposure to UroA enhances anticancer activity of adoptively transfer CD8^+^ T cells in colon cancer model (19). Similarly, we showed that UroA *in vitro* treatment in longer culture condition boosts CD8^+^ T cells antitumoral activity when adoptively transferred in B16 melanoma-bearing mice. Importantly, we revealed the accumulation of tumor-specific central memory T cells in the draining lymph nodes, known for their superior tumor control function (31, 32). Overall, these results highlight the translational potential of UroA supplementation for ACT product.

Although the safety profile of UroA has been tested in clinical trials (12, 15) and its property as mitophagy inducer extensively studied, the molecular target(s) have not been identified yet. Here, we showed that UroA reduces FOXO1 phosphorylation levels, promoting higher nuclear localization and the concomitant upregulation of key FOXO1 target genes such CD62L. Interestingly, UroA downregulates Akt phosphorylation, which controls FOXO1 transcriptional activity (38).

FOXO1 is known to regulate trafficking, naïve homeostasis, differentiation, and effector/memory response (7, 8), and mitochondrial biogenesis via PGC1α activity (43, 36). UroA has been described to induce mitophagy and to ameliorate mitochondrial metabolism in CD8^+^ T cells (19). Interestingly, we observed UroA-induced mitophagy only when T cells are culture IL15/7 condition, where the components of the mitophagy machinery are only present in response to IL15 and IL7 (50). While the UroA-induced mitophagy is cytokine dependent, the effect of UroA in increasing the expression of FOXO1 target genes is consistent across the different culture condition, suggesting the independency of mitophagy from FOXO1 activity. In fact, FOXO1 knockout T cells express comparable levels of mitophagy-related genes and display similar mitophagy activity (50).

To better distinct FOXO1 activity from mitophagy induction, genetic deletion of mitophagy machinery components could help to dissect this aspect (19). However, knocking out specific mitophagy target genes, such as Pink, Park2, Atg5, and Atg7 (19, 51, 52), impairs T-cell naïve homeostasis and dysregulated Akt and FOXO1 phosphorylation (53). The disrupted naïve T-cell homeostasis and activation will not reflect the initial condition where UroA immunomodulation take place and most probably will lead to erroneous conclusions. These technical limitations highlight the need for better model to investigate mitophagy and naïve T-cell function.

In conclusion, our data further demonstrate the antitumor activity of UroA supplementation and discover its efficacy in regulating naïve T cells and enhancing cancer immunosurveillance via FOXO1 activation.

## Supplementary Material

Figure S1 UroA supplementation does not modulate immune infiltrate composition

Figure S2 UroA improve functionality of T cells in vitro

Figure S3 UroA modulates FOXO1 target genes expression but not FOXO1 expression in vitro

Figure S4 UroA induces mitophagy in T cells culture in Il15/7 condition
