# Supplementary material for: Urolithin-A Promotes CD8+ T Cell–mediated Cancer Immunosurveillance via FOXO1 Activation
Source: Cancer Res Commun. 2024 May 3;4(5):1189–98. doi: 10.1158/2767-9764.CRC-24-0022 (PMC11067828; doi:10.1158/2767-9764.CRC-24-0022)
Supplement: Figure S1 — UroA supplementation does not modulate immune infiltrate composition [file crc-24-0022-s01.docx]

**Supplementary Figure S1**


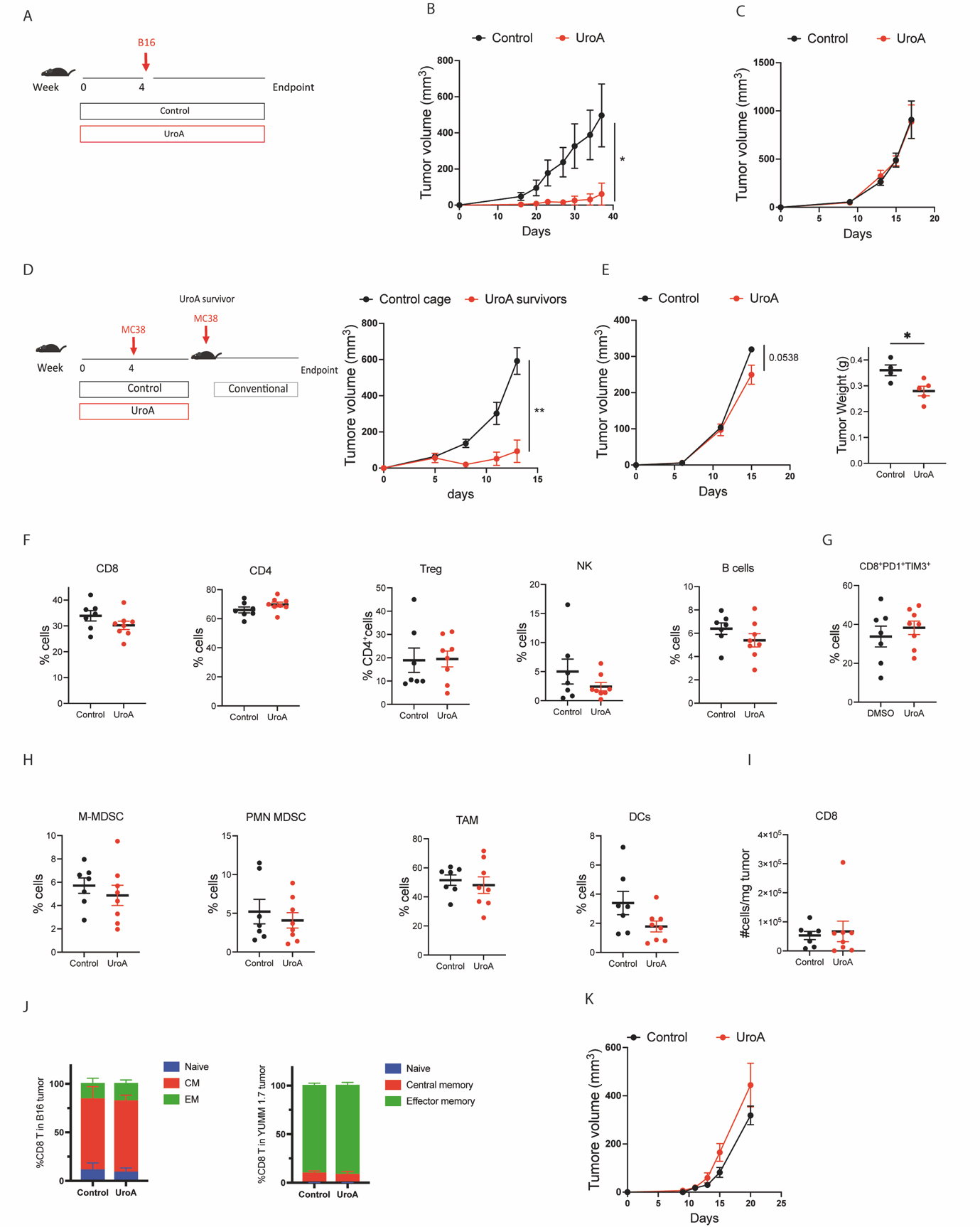


**Supplementary Figure S1 UroA supplementation does not modulate immune infiltrate composition**

(A) Schematic representation of experimental set up: B16 cells were subcutaneously injected in immunocompetent C57BL6 mice fed for four weeks with UroA enriched food. (B) Analysis of tumor growth rate of MC38 cancer cells subcutaneously engrafted in mice fed for four weeks with UroA enriched food. (C) Analysis of tumor growth rate of MC38 cancer cells subcutaneously engrafted in immunodeficient NSG mice fed for four weeks with UroA enriched food. (D) Tumor growth rate of MC38 cancer cells subcutaneously injected in control mice (control cage) and in survivor mice from B. (E) Tumor volumes of YUMM cancer cells subcutaneously engrafted in mice fed for four weeks with UroA enriched food(right). Tumor weight from E at day 15 post tumor cells injection (control n=4, UroA n=5). (F-G-H) Percentage of the indicated population in the immune infiltrate of B16 tumors from mice fed with control (n=7) or UroA enriched diet (n=8). (I) Number of CD8^+^ T cell in the TME of mice mice fed with control (n=7) or UroA enriched diet (n=8). (J) Frequency of naïve (CD62L^+^ CD44^-^) central memory CM (CD62L^+^CD44^+^) and effector memory EM (CD62L^-^CD44^+^) CD8^+^ Tcells in the TME of the indicated tumor. For B16 control n=4, UroA n=5, for YUMM 1.7 control n=7, UroA n=8. (K)Tumor growth rate of B16 cancer cells subcutaneously injected in mice receiving control or UroA diet the same day of tumor injection. Data are mean ± s.e.m., each dot represents a biological replicate. In figure B sample size= 7 mice. In figure C sample size n=5. In figure D control mice n=4, UroA survivors =3 mice. In figure E control=4, UroA=5. In figure K sample size n=5. Data were analyzed by two-sided student T (*=p<0.05,**=p<0.01). Representative results of at least two independent experiments or two pooled experiments.
