# Supplementary material for: Urolithin-A Promotes CD8+ T Cell–mediated Cancer Immunosurveillance via FOXO1 Activation
Source: Cancer Res Commun. 2024 May 3;4(5):1189–98. doi: 10.1158/2767-9764.CRC-24-0022 (PMC11067828; doi:10.1158/2767-9764.CRC-24-0022)
Supplement: Figure S2 — UroA improve functionality of T cells in vitro [file crc-24-0022-s02.docx]

**Supplementary Figure S2**


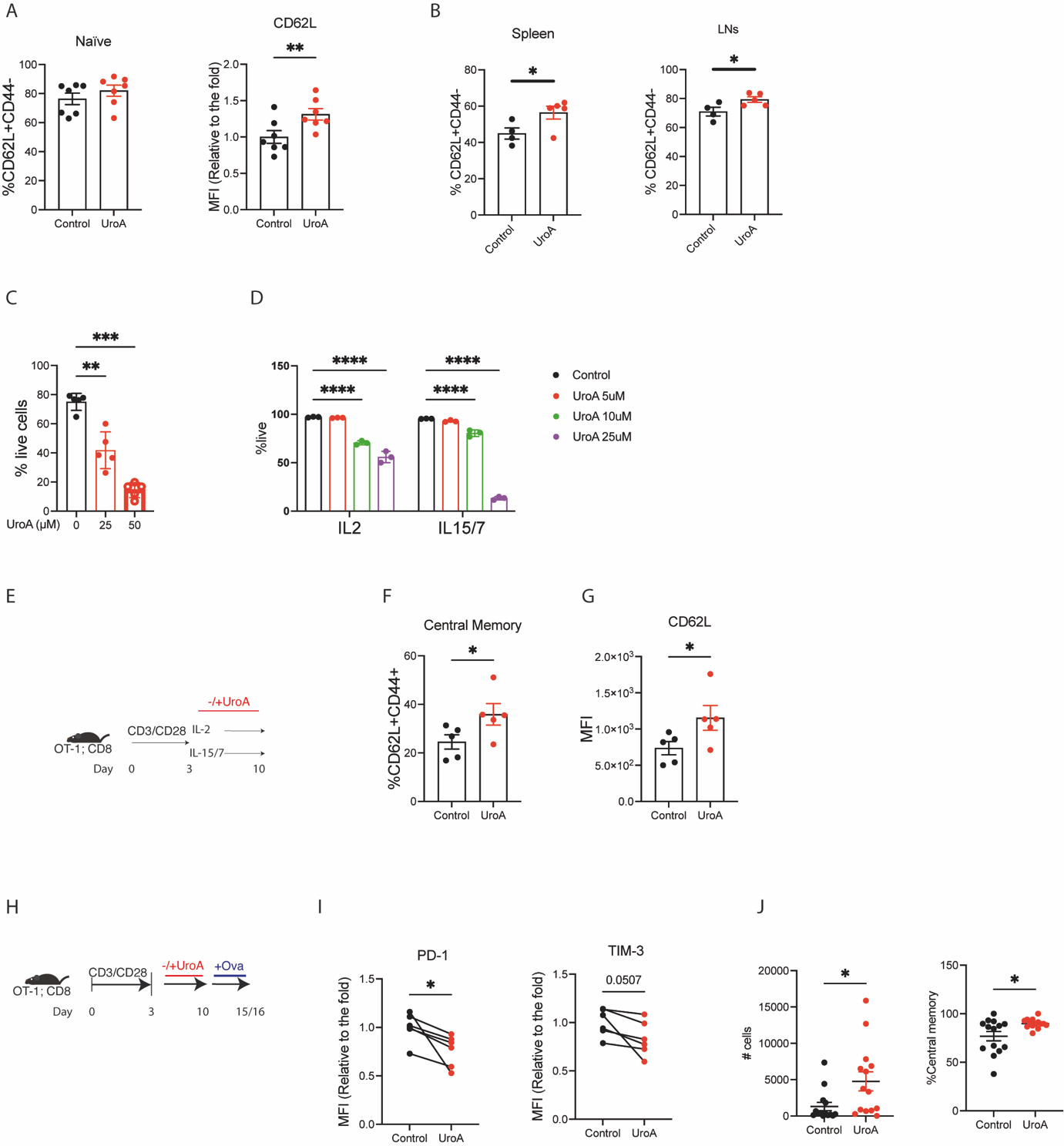


**Supplementary Figure S2 UroA improve functionality of T cells *in vitro***

(A) Frequency of naïve CD8^+^ T cells (CD62L^+^CD44^-^) isolated from the lymph nodes of mice fed with control or UroA enriched diet (left). Expression level of central memory marker CD62L in CD8^+^ T cells from A. (B) Frequency of naïve CD4^+^ T cells (CD62L^+^CD44^-^) isolated from the spleen and the lymph nodes (LNs) of mice fed with control or UroA enriched diet (C) Frequency of live CD8^+^T cells treated with the indicated UroA concentration for 72hours in presence of CD3/CD28 beads. (D) Frequency of live CD8^+^T cells treated with the indicated UroA concentration in IL-2 and IL-15/7 condition for 48 hours. (E) Schematic representation of *in vitro* culture system. (F) Frequency of Central Memory (CD62L+CD44+) in CD8^+^ T cells treated for 4 days with 5μM UroA in IL-2 condition *in vitro*. (G) Expression level of central memory marker CD62L in F. (H) Schematic representation of *in vitro* exhaustion model. (I) Expression levels of the indicated exhaustion markers in chronically stimulated cells as described (H). (J) Number and frequency of central memory (CD62L^+^CD44^+^) in draining lymph nodes derived from adoptively transferred control or UroA treated OT-1 CD8^+^T cells. Data are mean ± s.e.m. each dot represents a biological replicate. In figure A sample size n=7. In figure B control=4 , UroA =5. In Figure C sample size n=5. In figure D sample size n=3. In figures F and G sample size n=5. In Figure I sample size n=6. In figure J sample size n=14. Data were analyzed by two-sided student T test (*=p<0.05,**=p<0.01). Representative results of two or three independent experiments or pooled experiments.
