# Supplementary material for: Urolithin-A Promotes CD8+ T Cell–mediated Cancer Immunosurveillance via FOXO1 Activation
Source: Cancer Res Commun. 2024 May 3;4(5):1189–98. doi: 10.1158/2767-9764.CRC-24-0022 (PMC11067828; doi:10.1158/2767-9764.CRC-24-0022)
Supplement: Figure S3 — UroA modulates FOXO1 target genes expression but not FOXO1 expression in vitro [file crc-24-0022-s03.docx]

**Supplementary Figure S3**


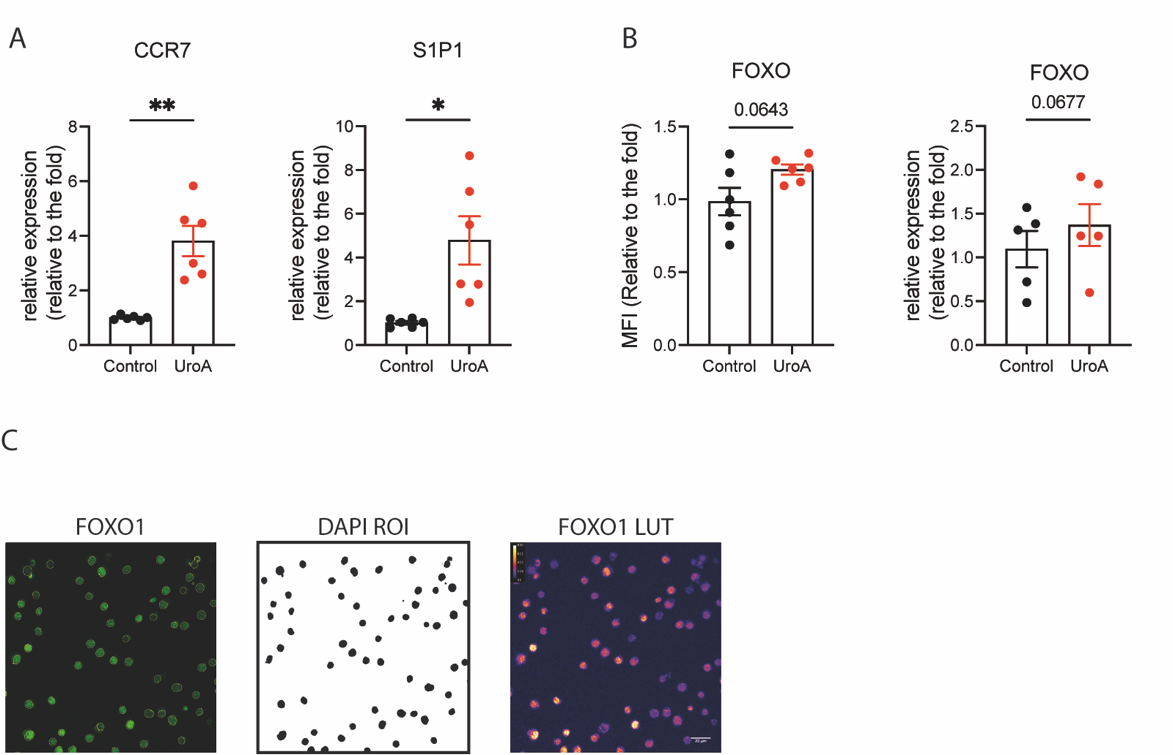


**Supplementary Figure S3 UroA modulates FOXO1 target genes expression but not FOXO1 expression *in vitro***

(A) Quantification of FOXO1 target genes in CD8^+^ T cells culture with 5μM UroA in IL2 condition *in vitro* (B) FOXO1 expression level in CD8^+^ T cells culture for 48 hours with 5μM UroA in presence of IL15 and Il17 cytokines (left) *in vitro*. Expression level of FOXO1 mRNA in CD8^+^ T cells culture with for 48 hours with 5μM UroA in presence of IL2 *in vitro* (right). (C) Representative images for FOXO1 nuclear analysis. FOXO1 intensity was calculated in the region defined automatically by a mask (middle) that includes all area with Dapi fluorescence. Left: FOXO1 staining. Middle: DAPI Mask. Right: FOXO LUT image. In figure A sample size n=6. In figure B sample size n=6 and n=5. Data are mean ± s.e.m., each dot represents a biological replicate. Data were analyzed by two-sided student T test (*=p<0.05, **=p<0.01)[1]. Results of two independent experiments.
