# Supplementary material for: Urolithin-A Promotes CD8+ T Cell–mediated Cancer Immunosurveillance via FOXO1 Activation
Source: Cancer Res Commun. 2024 May 3;4(5):1189–98. doi: 10.1158/2767-9764.CRC-24-0022 (PMC11067828; doi:10.1158/2767-9764.CRC-24-0022)
Supplement: Figure S4 — UroA induces mitophagy in T cells culture in Il15/7 condition [file crc-24-0022-s04.docx]

**Supplementary Figure S4**


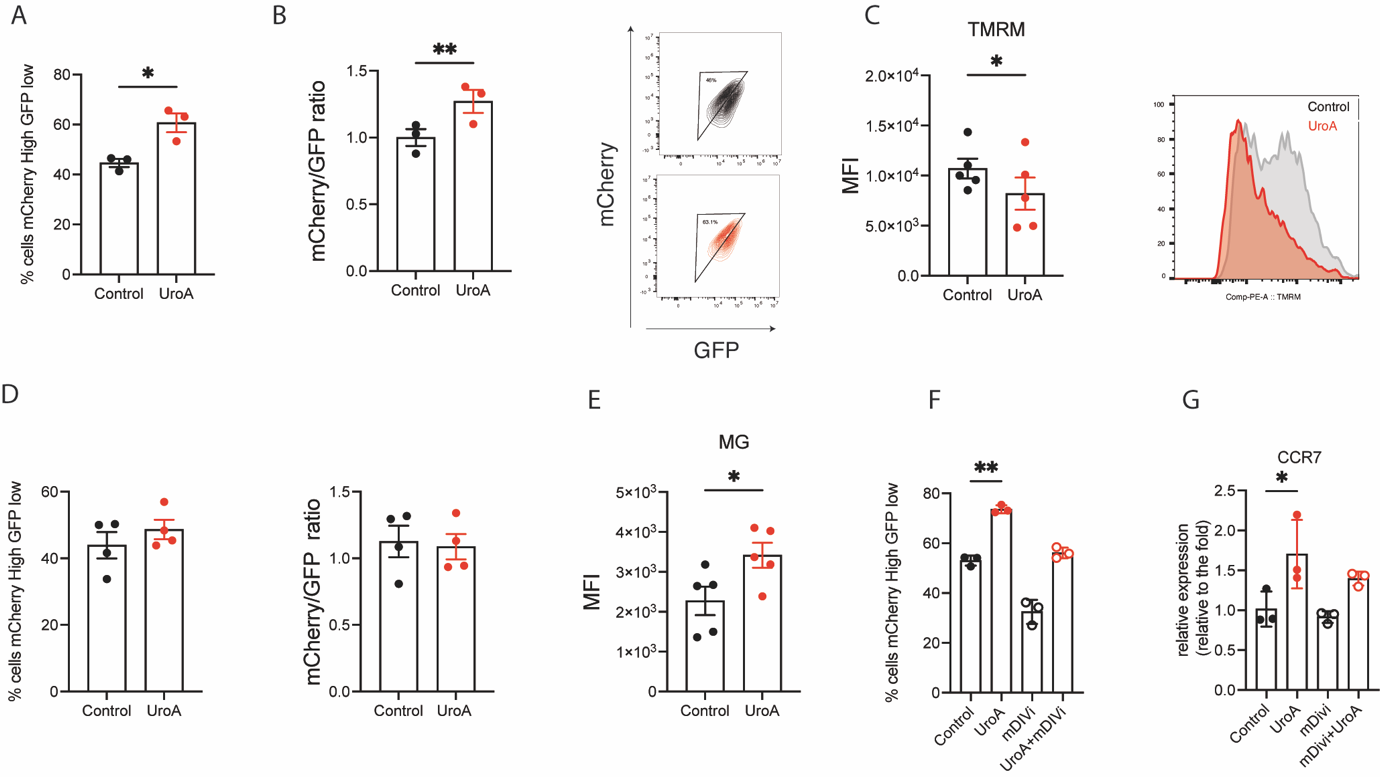


**Supplementary Figure S4 UroA induces mitophagy in T cells culture in Il15/7 condition**

(A) Quantification of Mito-QC CD8^+^ T cells engaging mitophagy under indicated condition (UroA 5μM for 24 hours) *in vitro* in IL15/7condtion. The higher gating (mCherry high GFP low) represents cells undergoing high rate of mitophagy. (B) Quantification of mCherry/GFP MFI ratio in CD8^+^ T cells from A. (C) Quantification of mitochondrial membrane potential (revealed by TMRM staining) in CD8^+^ T cells treated *in vitro* with UroA. (D) Quantification of Mito-QC CD8^+^ T cells engaging mitophagy under indicated condition (UroA 5μM for 24 hours) *in vitro* in IL2 condition. The higher gating (mCherry high GFP low) represents cells undergoing high rate of mitophagy (left). Quantification of mCherry/GFP MFI ratio in CD8^+^ T cells from D. (E) Quantification of mitochondrial mass (revealed by mitotracker MG staining in CD8^+^ T cells treated for 48 hours with UroA (5μM) in IL15/7 condition *in vitro*. (F) Quantification of Mito-QC CD8^+^ T cells engaging mitophagy under indicated condition. (G) Quantification of *CCR7* in CD8^+^ T treated with UroA(5μM) and mDdivi (10μM) *in vitro*. Data are mean ± s.e.m., each dot represents a biological replicate. In figures A and B sample size n=3. In figures C,D and E sample size n=5. In figures F and G samples size n=3. Data were analyzed by two-sided student T test or ANOVA test followed by multiple comparison test (*=p<0.05, **=p<0.01). Representative results of at least two independent experiment.
